# No effect of anti-TNF-α agents on the surgical stress response in patients with inflammatory bowel disease undergoing bowel resections: a prospective multi-center pilot study

**DOI:** 10.1186/s12893-018-0425-0

**Published:** 2018-11-03

**Authors:** Alaa El-Hussuna, Niels Qvist, Marie Strøm Zangenberg, Anne Langkilde, Volkert Siersma, Sara Hjort, Ismail Gögenur

**Affiliations:** 1 Department of Surgery, Aalborg University Hospital, Hobrovej 18-22, 9000 Aalborg, Denmark; 2 Department of Surgery, Odense University Hospital, Odense, Denmark; 3 Department of Surgery, Slagelse Hospitals, Slagelse, Denmark; 4 Copenhagen University Hospital Hvidovre, Optimed, Clinical Research Centre, København, Denmark; 5 The Research Unit for General Practice and Section of General Practice, Department of Public Health, University of Copenhagen, Copenhagen, Denmark; 6 Center for Surgical Science, Department of Surgery, Zealand University Hospital, Roskilde, Denmark

**Keywords:** Anti-TNF alpha, Surgical stress response, Wound healing, Inflammatory bowel disease, Crohn’s disease, Anastomotic leak, Ulcerative colitis, Interleukins

## Abstract

**Background:**

TNF-α plays a role in angiogenesis and collagen synthesis, both essential in the wound healing process. There are concerns that pre-operative anti-TNF-α treatment may influence the surgical stress response and increase the risk of surgical complications. The aim of this study was to describe the surgical stress response in patients with inflammatory bowel disease (IBD) and to investigate whether the pre-operative administration of anti-tumor necrosis factor alpha (anti-TNF-α) agents modify the surgical stress response.

**Methods:**

This was a prospective, multi-center cohort pilot study. The primary outcome was the change in concentration of immunological biomarkers of the surgical stress response (TNF-α, IL-6, and IL-10). Secondary outcome measures were changes in IL-8, IL-17A, C-reactive protein, white blood cells, cortisol, transferrin, ferritin, and D-Dimer in addition to 30 days’ post-operative complications and length of post-operative stay in the hospital (LOS).

**Results:**

Forty-six patients with IBD undergoing major abdominal surgery were included, and 18 received anti-TNF- α treatment pre-operatively. Peak increase of most of the immunological biomarkers occurred 6 hours after surgical incision. Then the concentration decreased after 24 h followed by a plateau at 48 h. After adjusting for confounders including detectable blood concentrations, no difference in the concentrations of immunological, endocrinological or haematological biomarkers of stress was found between anti-TNF-α treated and anti-TNF-α naïve patients. No increase in post-operative complications or LOS was noticed in patients who received anti-TNF-α treatment.

**Conclusions:**

Anti-TNF-α did not affect surgical stress response in this pilot study. Withdrawal of anti-TNF-α drugs prior to surgical intervention in IBD patients might not be justified without measurement of drug concentration and drug antibodies.

**Trial registration:**

Clinicaltrails.gov.: NCT01974869.

## Background

Surgical injury induces a stress response with activation of endocrine, metabolic, and immunologic mediators aiming to restore hemostasis and induce tissue repair. The immunologic and inflammatory response to stress is regulated by cytokines produced in activated macrophages, fibroblasts, endothelial cells, and giant cells. Tissue injury and infection are sensed by a group of protein receptors called pattern recognition receptors (PRRs) that can be activated by pathogen-associated molecular patterns (PAMPs) and damage-associated molecular patterns (DAMPs). A wide range of PRRs have been described, including membrane bound Toll-like receptors (TLRs) and C-type lectin receptors (CLRs), or cytoplasmic receptors such as NOD (Nucleotide-binding oligomerisation domain)-like receptors (NLR). Following stimulation of these receptors, multiple downstream proteins will be activated leading to intracellular signaling pathways that culminate in activation (phosphorylation) of transcription factors such as NF-kB among others which in turn drive the production of a large array of both pro- and anti-inflammatory cytokines and chemokines [1, 2].

The cytokines of special interest include interleukins (IL) and tumor necrosis factor-alpha (TNF-α).

Anti-TNF-α agents (biologicals) are antibodies directed against this key cytokine acting in two ways: First, by scavenging soluble TNF-α, thereby preventing activation of immune cells via TNF-a-receptors; and second, by “reverse signaling” acting on membrane bound TNF-α -receptors on monocytes and T-cells inducing apoptosis and inhibition of further cytokine release [1].

Besides being an important component of the immune defense, TNF-α plays a role in angiogenesis [2], collagen synthesis [3–5] and wound healing. Inhibition of these pathways may impair wound healing after surgery. Several observational retrospective studies have been carried out to investigate the risk of post-operative complications in patients with IBD who received pre-operative anti- tumor necrosis factor-alpha (anti-TNF-α) treatment [6–8]. The results of these studies are conflicting but a common point is that at least 1/3 of patients received anti-TNF-α) treatment [8].

The aim of this study was to describe the surgical stress response in patients with IBD undergoing surgical intervention and to investigate whether anti-TNF-α agents modify the surgical stress response.

## Methods

### Study design

The null hypothesis was that pre-operative administration of anti-TNF-α agents within 12 weeks before surgery, have no significant effect on surgical stress response. To investigate this; a prospective, non-interventional multi-center pilot study was designed.

*The primary outcome* measure was the difference in the plasma concentrations of the main immunological biomarkers of surgical stress response (TNF-α, IL-6, and IL-10) between anti-TNF-α treated patients and anti-TNF-α naive.

*The secondary outcome measures* were difference in the plasma concentrations of other biomarkers of surgical stress including IL-8, IL-17A, the ratio of TNF-α/ IL-10 and Il-6/IL10, cortisol, transferrin, ferritin, and D-Dimer in addition to 30-days, post-operative complications and length of hospital stay (LOS). Overall complication was defined as any deviation from the expected post-operative recovery. Intra-abdominal septic complications (IASC) were defined as overt anastomotic leakage, intra-abdominal abscess formation or enteric fistula. Superficial surgical site infection (SSI) was defined as clinically documented skin infection at the site of surgery with or without positive culture. Grade of complications were assessed using Clavian-Dindo classification of surgical complications.

The choice of sampling intervals at six, 24 and 48 h after surgical incision was based on previous investigations [9–13]. Biomarkers of surgical stress were selected according to the existing evidence [2, 5, 9, 11–24].

Inclusion criteria: adult patients with Crohn’s disease (CD) or ulcerative colitis (UC) who were scheduled to elective intestinal resection or terminal stoma closure in three Danish university hospitals during the study period (March 2014–May 2016). Open as well as laparoscopic approaches were included.

Exclusion criteria: patients with pre-operative sepsis, acute intestinal obstruction, patients operated in acute setting (within 48 h of admission) and patients who had loop ileostomy take down without laparotomy or laparoscopy.

### Details of the procedures

#### Laboratory procedures

Peripheral blood samples were taken before the induction of anesthesia, and six, 24 and 48 h after surgical incision. EDTA plasma and serum was separated by centrifugation, aliquoted and stored at − 80 °C until analysis.

The concentration of anti-TNF-α biological compounds administered pre-operatively (drug concentration) was measured in peripheral blood at the day of surgery together with antibodies against the specific compound (anti-drug antibodies). Details of the method used explained in the laboratory homepage [25].

Cortisol was measured by ELISA (DRG International, Inc.; Catalog number: EIA 1887; Marburg, Germany). IL-6, IL-10, IL-17A, and TNF-α were measured by a human high sensitive magnetic ProCartaPlex luminex kit (eBioscience; Catalog number: EPX040–00000-801; Vienna, Austria). IL-8 and D-Dimer were measured using ProCartaPlex Human IL-8 simplex, ProCartaPlex Human D-Dimer simplex, and Human Basic kit (eBioscience; catalog numbers: EPX010–10204-901, EPX010–12149-901, and EPX010–10420-901; Vienna, Austria). All samples were measured in duplicates according to the manufactures instructions, using the mean for statistical analyses. Plasma levels of CRP, transferrin, ferritin and D-dimer were measured using standard methods by the Department of Clinical Biochemistry, Copenhagen University Hospital, Amager and Hvidovre, Denmark.

#### Anesthesia, surgery and post-operative care

All the operations took place between 08:00 a.m. - 04.00 p.m. to avoid circadian rhythm as a confounder. General anesthesia was administered according to the standard practice of the anesthesia department in the participating hospitals. All patients received single prophylactic pre-operative antibiotics at the induction of anesthesia. The type and dose was determined by local standard of pre-operative care in the participating hospitals. Laparoscopic surgery and enhanced post-operative recovery principles were the standard procedures in the participating centers.

### Statistical analysis

#### Sample size

Reference values for the changes in the biomarkers for surgical stress in IBD patients were not available at the time of the study to allow precise sample size calculations. Chalhoub et al. showed that 28 patients were needed to demonstrate a significant change in TNF-α concentration after moderately stressful surgery [26]. Moreover, Dimopoulou et al. [14] found that 40 patients should be included to detect a significant correlation between the values in TNF-α concentration and post-operative complications. Based on these two studies (non-IBD patients) and a meta-analyses by the authors [8], this pilot study was a priori designed to recruit at least 40 patients of whom 1/3 had received anti-TNF-α treatment prior to surgery keeping in mind that repeated measures will reduce the expected variations in outcome

#### Statistical methods

Pre-operative and peri-operative characteristics were compared between anti-TNF-α treated and anti-TNF-α naïve patients with chi-squared tests. Median, inter-quartile range (IQR), minimum and maximum were used to visualize changes in the concentration of biomarkers from baseline (before operation) to one of the post-operative follow-up time-points (six, 24 and 48 h after surgical incision). The difference of the medians of the changes from baseline between anti-TNF-α treated and anti-TNF-α naïve patients was assessed using a bootstrap approach in which patients (retaining the up-to-four-measurements per patient) were sampled with replacement using 1000 bootstrap replicates. To reduce bias from confounding, the calculated medians were weighted by a propensity score, i.e. the inverse of the estimated probability of the received anti-TNF-α regimen conditional on potential confounders. These probabilities were estimated from a multivariable logistic regression model included: Harvey-Bradshaw index for disease severity, nutritional risk screening score, parenteral nutrition, previous IBD-related abdominal operations, steroid stress dose, pre-operative Dexamethasone, epidural analgesia, access to abdominal cavity (open versus laparoscopic), type of resection and disease classification in case of CD. These were pre-operative factors that were either significantly different between the two anti-TNF-α regimens, or deemed important determinants from clinical experience. Propensity scores were re-calculated within each bootstrap replicate.

A permutation test, in which the four measurements for each patient were randomly redistributed over the time points in 1000 replicates to assess the null hypothesis of no development, was used to assess the statistical significance of the changes in the median concentration of biomarkers over time.

Logistic regression was used to investigate post-operative outcome where adjustment for confounding was done by stepwise backwards elimination, starting with a model including all pre- and peri-operative characteristics deemed clinically and/or statistically significant different between the two treatment groups. Variables were then removed one by one until all variables had *p* < 0.10.

All analyses, except for the bootstrap analyses, were done using IBM SPSS Statistics for Windows, Version 19.0. Armonk, NY: IBM Corp.2010. The bootstrap analyses were performed in the R environment for statistical computing version 3.1.2. A significance level of 5% was chosen.

We used non-parametric, robust methods for inference, i.e. a non-parametric bootstrap on the difference of propensity score weighted medians. These methods are described in detail in the statistics section of the paper. The SAMPLE guidelines were not very applicable to the inferential methods we used. This has been referred to in the manuscript.

## Results

Patients were identified using outpatient’s clinic records, operation lists and reports from IBD conferences. All patients who fulfilled the inclusion criteria accepted to participate in the project. One patient was excluded because blood samples were accidently discarded. This explorative study succeeded, thus, in recruiting 46 patients, of which 18 had one type or another of anti-TNF-α agent treatment within 3 months prior to surgery.

### Background characteristics

Median age was (42.5, IQR 23) years, 25/46 of the patients were females (54.3%). Median body mass index was (23.5, IQR 6.3). In anti-TNF- α group, 4/18 (22.2%) had one or more co-morbidities compared to 7/28 (31.8%) in anti-TNF-α naïve. No difference in the mean duration of disease was found between anti-TNF-α treated patients (7.61 SD ± 7.82) years and anti-TNF-α naive (10.11 SD ± 9.15). Pre-and intra-operative patients’ characteristics are reported in Table 1.

**Table 1:** Pre-operative and intra-operative patients’ characteristics in 46 IBD patients treated with anti-TNFα compared to anti-TNFα naive

Patients’ characteristics	Anti-TNFα treat. 18/46 (39.1%)	Anti-TNFα naïve 28/46 (60.9%)	Uni-variate
Age (mean ± SD)	38.7 ± 16.36	44.39 ± 12.62	*ns*
Female	11/18 (61.1%)	14/28 (50%)	*ns*
Body mass index	24.93 (±SD 6.12)	24.7 (±SD 5.37)	*ns*
Type of disease *n* (%)			*ns*
Crohn’s disease	13/18 (72.2%)	19/28 (67.9%)	
Ulcerative colitis	5/18 (27.8%)	9/28 (32.1%)	
Smoking *n* (%)			*ns*
Non-or ex-smoker	14/18 (77.8%)	22/28 (78.6%)	
Smoker	4/18 (22.2%)	6/28 (21.4%)	
Steroids *n* (%):	7/18 (38.9%)	9/28 (32.1%)	*ns*
Immuno-modulators *n* (%):	8/18 (44.4%)	7/28 (25%)	
NSAID intake preoperative *n* (%):	0	1/28 (3.6%)	*ns*
Anti-Coagulant intake *n* (%):	1/18 (5.6%)	0	*ns*
Harvey-Bradshaw Index in CD patient more than median (7.5) *n* (%):	4/18 (36.4%)	8/28 (61.5%)	*ns*
Pre-operative Albumin mmol/l (mean ± SD)	33.22 ± 7.53	36.89 ± 4.36	*ns*
Pre-operative Haemoglobin mmol/l (mean ± SD)	7.86 ± 0.94	8.09 ± 1.01	*ns*
Nutritional risk screening score *n* (%)			*ns*
No risk	6/18 (33.3%)	20/28 (71.4%)	
Mild	6/18 (33.3%)	4/28 (14.3%)	
Moderate	3/18 (16.75)	2/28 (7.1%)	
Sever	3/18 (16.7%)	2/28 (7.1%)	
Pre-operative parenteral nutrition *n* (%):	5/18 (27.8%)	1/28 (3.6%)	p = 0.028
Steroid stress dose *n* (%):	5/18 (27.8%)	0	p = 0.003
Dexamethasone *n* (%)			*ns*
4 mg	1/18 (5.6%)	1/28 (3.6%)	
8 mg	2/18 (11.1)	9/28 (32.1%)	
Pre-operative Epidural analgesia *n* (%):	5/18 (27.8%)	7/28 (25%)	*ns*
Access to abdomen *n* (%)			*ns*
Laparoscopic	13/18 (72.2%)	17/28 (60.7%)	
Converted	3/18 (16.7%)	2/28 (7.1%)	
Open	2/18 (11.1%)	9/28 (32.15%)	
Type or resection *n* (%)			*ns*
SM & IC	9/18 (50%)	11/28 (39.3%)	
Colectomy and/or Rectal	8/18 (44.4%)	12/28 (42.9%)	
Stoma closure	1/18 (5.5%)	5/28 (17.8%)	
Post-operative Epidural analgesia *n* (%):	6/18 (33.3%)	12/28 (42.9%)	*ns*
Post-operative NSAID used *n* (%):	0	2/28 (7.1%)	*ns*
Post-operative parenteral nutritional support *n* (%):	5/18 (27.8%)	1/28 (3.6%)	*p* = 0.028

All operations were performed with a specialist surgeon in charge. *Anti-TNF-α* Anti-tumour necrosis factor drugs, *Ns* non-significant, *SM* small bowel, *IC* Ileo-colic

Anti-TNF-α naïve patients had higher rate of previous IBD surgeries compared to anti-TNF- α treated patients (*p* = 0.003). Anti-TNF-α treated patients were more likely to receive pre-operative parenteral nutritional support (*p* = 0.028). Moreover, anti-TNF-α treated patients with CD had a longer ileocecal/ileo-colic resected segment (mean 31.11 SD ± 35.51) cm versus 27.43 SD ± 18.83 *p* = 0.036) and were more likely to suffer from a stricturing CD phenotype (10/76.9% versus 8/42.1% *p* = 0.01). But this did not affect the results in multivariate analyses. Type of surgical incision and type of bowel resections were similar in the two groups.

In the 18 patients who received anti-TNF-α pre-operatively, different type of anti-TNF-α drugs were administered using different doses with wide variations of the interval from last administered dose to surgery (Table 2). Thus, 44% of anti-TNF-α treated patients had undetectable drug concentration in peripheral blood and only three of these 18 patients had anti-drug antibodies at the time of surgery.

**Table 2:**
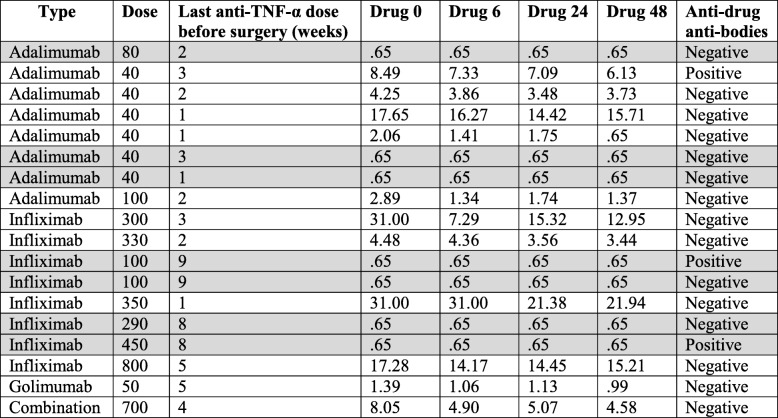
Type of anti-TNF-α agents, duration of treatment, drug concentration and presence of anti-drug antibodies

Drug 0: drug concentration μg/mL before surgery. Drug 6: drug concentration μg/mL 6 h after surgery. Drug 24: drug concentration μg/mL 24 h after surgery. Drug 48: drug concentration μg/mL 48 h after surgery

Concentration of 0.65 μg/mL refers to undetectable drug concentrations (gray shadowed). Techniques used to measure Anti-drug antibodies are mentioned in the website of laboratory (http://www.wieslab.com/diagnostic-services/index.php?langId=1&headId=72&subId=143&pageId=195)

### Pattern of change in surgical stress markers in IBD

**Fig. 1:**
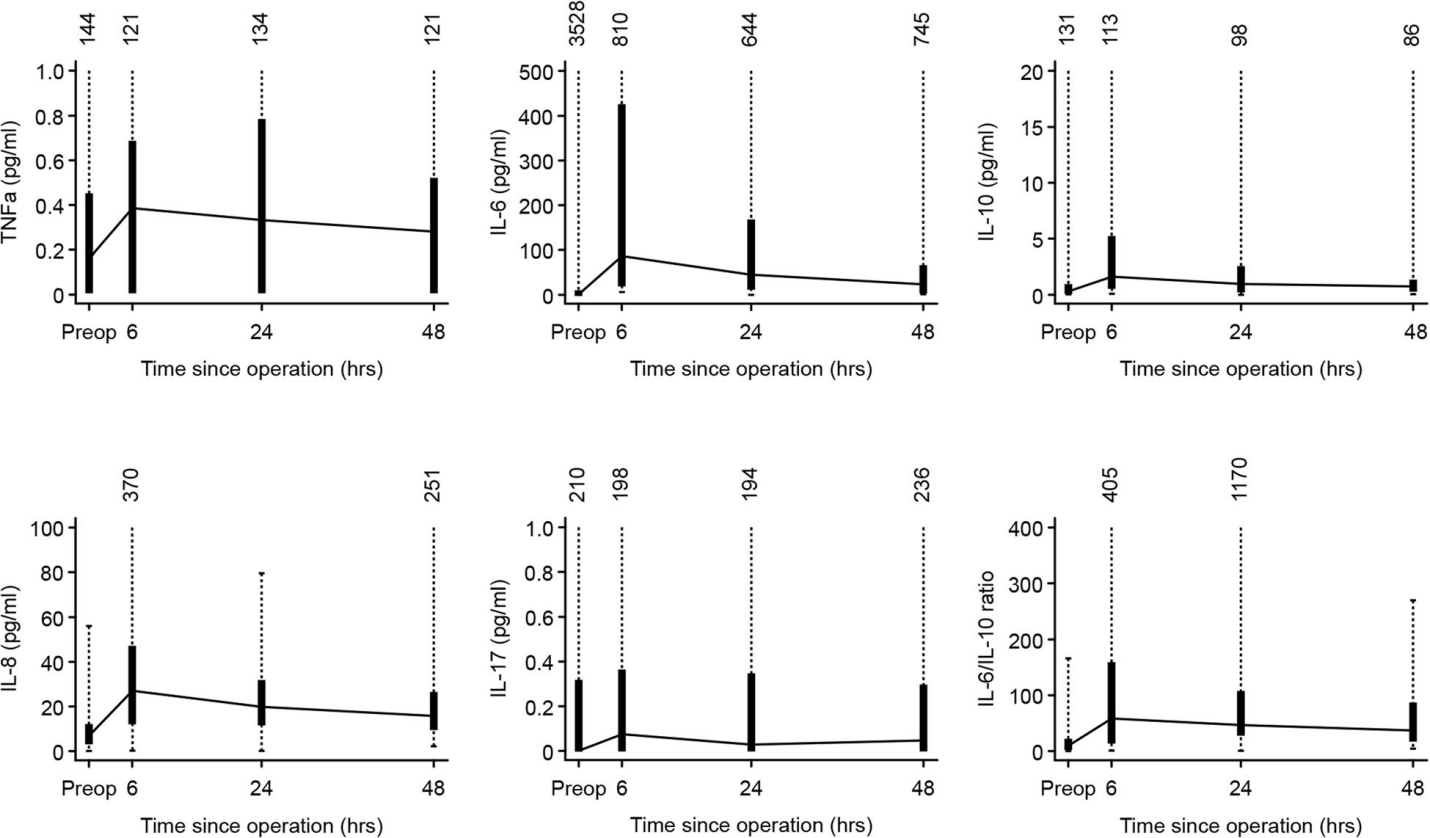
Surgical stress response in 46 patients with inflammatory bowel disease who underwent surgical interventions as part of disease treatment. Main immunological biomarkers of stress are shown. The box shows the median and inter-quartile while the numbers above show the concentrations for outliers

**Fig. 2:**
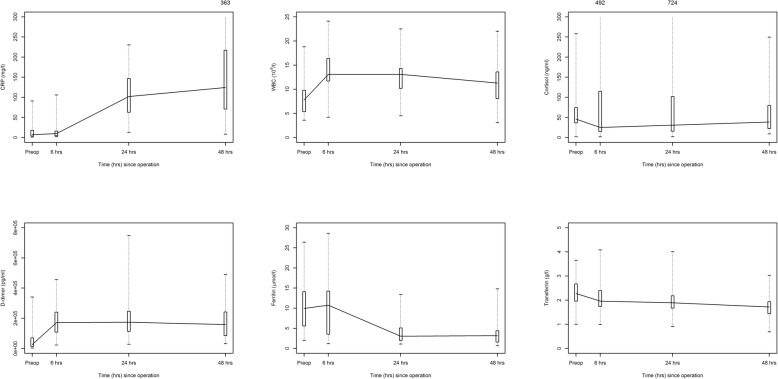
Surgical stress response in 46 patients with inflammatory bowel disease who underwent surgical interventions as part of disease treatment. Immunological, endocrinological and hematological biomarkers of stress are shown. The box shows the median and inter-quartile while the numbers above show concentrations for the outliers

Figures 1 & 2 depict the surgical stress response according to all 12 inflammatory biomarkers. Peak increase in TNF-α occurred 6 hours after surgical incision (median 0.596, IQR range 0.679) then the concentration decreased after 24 h (median 0.517, IQR range 0.969) followed by a plateau at 48 h (median 0.446, IQR range 0.655). The same pattern was observed for IL-6, IL-8, IL-10, IL-17A, IL-6/IL-10 ratio, WBC, D-Dimer, ferritin and transferrin while CRP peaked at 48 h after surgical incision. The ratio of TNF- α/IL-10 and cortisol decreased at 6 hours then started to increase at 24 h reaching a plateau at 48 h. The stress response over time was significant in all biomarkers (*p* < 0.01) except TNF-α, IL-17A and cortisol.

### Difference in surgical stress response between the two groups

**Fig. 3:**
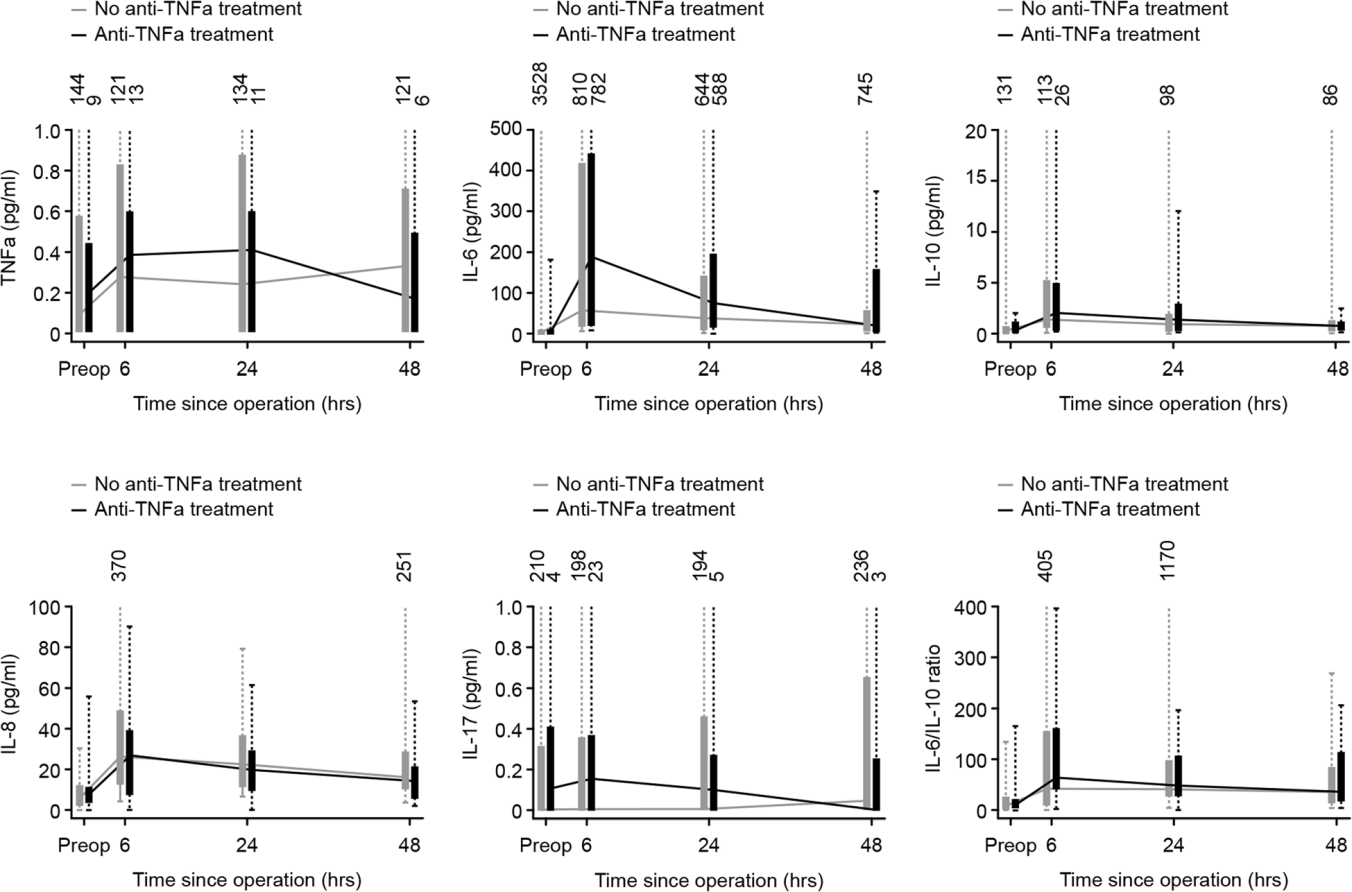
Surgical stress response in patients treated with anti-TNF-α agents versus anti-TNF-α naive. The figure shows only the main immunological biomarkers of stress. The box show the median and inter-quartile while the numbers above show the concentrations for outliers

There was a tendency towards a higher concentration in most of the inflammatory biomarkers in patients treated with anti-TNF-α agents compared to the anti-TNF-α naive as shown in Fig. 3. This was more pronounced in patients with detectable drug concentration and no anti-drug antibodies. However, the differences were not statistically significant (Table 3).

**Table 3:** The effect of anti-TNF-α treatment on surgical stress response

Biomarkers of stress	Change from baseline to 6 h.		Change from baseline to 24 h.		Change from baseline to 48 h.		Change from 6 to 24 h.	
	median (95% CI)	*p*	median (95% CI)	*p*	median (95% CI)	*p*	median (95% CI)	*p*
TNF-α	−0.25 (− 3.54; 0.24)	0.49	−0.20 (−1.97; 0.23)	0.44	0.00 (− 0.19; 2.42)	0.37	0.00 (0.00; 0.16)	0.04
IL-6	− 360.46 (− 668.19; 37.01)	0.13	− 200.86 (− 498.45; 29.67)	0.11	− 139.98 (− 276.06; 7.68)	0.12	3.87 (− 62.26; 163.41)	0.84
IL-10	− 0.97 (− 2.52; 1.34)	0.40	−1.29 (− 8.00; 0.17)	0.10	−0.03 (− 1.06; 0.64)	0.88	− 0.25 (− 1.08; 1.16)	0.40
IL-8	2.79 (− 12.25; 27.16)	0.56	5.25 (− 8.90; 17.55)	0.44	5.95 (− 6.83; 11.30)	0.48	−1.10 (−9.10; 7.80)	0.76
IL-17A	0.00 (− 0.03; 0.58)	0.08	0.00 (−1.13; 0.08)	0.70	0.00 (0.00; 0.15)	0.02	0.00 (−2.45; 0.10)	0.14
TNF-α/IL-10 ratio	−0.02 (− 0.30; 1.36)	0.96	0.03 (− 0.10; 3.10)	0.39	0.03 (− 0.15; 0.87)	0.33	0.02 (− 0.12; 0.08)	0.66
IL-6/IL-10 ratio	− 100.15 (− 151.21; 30.79)	0.13	−4.87 (− 145.59; 33.27)	0.59	−72.65 (− 123.24; 6.39)	0.13	18.09 (− 36.61; 63.46)	0.23
WBC	−0.09 (− 4.55; 3.66)	0.97	− 0.01 (− 3.95; 6.31)	0.78	0.27 (− 3.80; 5.82)	0.65	0.25 (−2.75; 4.70)	0.23
CRP	0.00 (− 4.14; 4.59)	0.88	−46.90 (− 76.10; 26.30)	0.22	−104.64 (− 179.39; 76.25)	0.39	−8.45 (− 55.20; 27.11)	0.62
Cortisol	23.80 (− 266.92; 110.51)	0.37	32.01 (− 10.86; 118.55)	0.20	−17.41 (− 137.82; 89.21)	0.51	9.04 (− 6.33; 38.60)	0.26
D-Dimer	−11.14 (− 110.31; 92.14)	0.68	28.59 (− 122.81; 80.70)	0.84	50.64 (− 126.71; 129.29)	0.61	5.95 (− 39.99; 69.11)	0.57
Ferritin	−2.20 (−6.58; 3.50)	0.48	− 0.35 (− 5.08; 3.92)	0.75	−1.33 (− 6.56; 2.98)	0.26	0.60 (− 6.31; 5.60)	0.91
Transferrin	−0.02 (− 0.23; 0.21)	0.93	−0.06 (− 0.30; 0.13)	0.4	−0.20 (− 0.40; 0.09)	0.15	0.02 (− 0.14; 0.11)	0.82

Reported in the table are the differences of the medians of the stress markers between anti-TNF-α naive and anti-TNF-α treated patients. Adjustment for confounding was done by propensity score weighting of the medians. 95% confidence intervals (95%CI) and *p*-values are estimated using a bootstrap approach

*P: refers to p value*

### Sub-group analyses

Sub-group analyses was done by selecting patients who underwent laparoscopic ileocecal/ileo-colic resections (12/46) to obtain a homogenous group of patients with same type of surgical procedure. Then by comparing anti-TNF-α treated and anti-TNF-α naïve in this sub-group showed no significant difference in surgical stress response (results not shown).

### Post-operative outcome

No difference was found in adjusted analyses of overall complications between the two groups (27.8% versus 28.6%), superficial SSI (7.8% versus 7.1%), IASC (5.6% versus 7.1%) and re-admission rates (22.2% versus 25%). Mean LOS was 5.33 (± 2.57) for anti-TNF- α treated versus (6.25 ± 3.01) in the anti-TNF- α naive group. The difference was not statistically significant. The time interval between last dose of anti-TNF- α and the day of surgery did not affect the rate of complications. Nor did the type of anti-TNF- α agents used (Table 2).

## Discussion

This explorative prospective multi-center pilot study described the pattern of surgical stress response in IBD patients as measured by evidence-based biomarkers of surgical stress response. The results showed that the administration of anti-TNF-α drugs in the pre-operative period did not have a significant effect on the immunological, hematological or endocrinological biomarkers of the surgical stress response. At first glance, this might be unexpected as the anti-TNF-α are potent drugs that affect the immune system and thus the inflammatory phase of wound healing. However, deeper insights reveal that this is not a stand-alone observation because the effect of anti-TNF- α drugs on surgical site infection and anastomotic leak was not significant in two nation-wide database studies [27, 28] and four experimental studies in which confounders were well controlled and measurements of effective drug concentration were available [29–32]. Moreover, Lau et al. showed a correlation between anti-TNF-α drugs concentration of more than 3 μg/mL and post-operative infectious complication but no wound healing related complications in CD patients [33]. The measurement of detectable blood concentration of anti-TNF-α might explain this because-as shown in this cohort- not all patients who received anti-TNF-α had a detectable drug concentration in their blood prior to surgery and some of them had neutralizing antibodies. This finding may also explain some of the controversies seen in observational studies [8].

However, having undetectable drug concentration do not rule out that anti-TNF-α drugs had an effect on immune system.

Anti-TNF-α drugs did not reduce the concentration of TNF-α in our study. A finding that is in line with two other studies [34, 35], in which serial measurements of 17 serum cytokines were conducted in 37 and 24 patients respectively. This can be related to a dose-dependent effect in which the anti-TNF-α drugs reduce the concentration of TNF-α in higher doses. Verification of such hypothesis as well as finding the cut-off concentration are to be investigated in experimental studies and/or in clinical studies where the interval between anti-TNF-α administration and surgery is not more than few days.

We did not find an association between anti-TNF-α treatment and post-operative outcomes. This did not appear to be explained by possible clinically and intra-operative factors that might influence the stress response to surgery or drug concentration and presence of anti-drug antibodies, as we adjusted for these. However, this might be due to small sample size [8] or due to a dose-dependent effect of anti-TNF-α on post-operative outcome [33].

Limitations of this study include the small sample size (pilot study) and the lack of measurements of other influential factors in wound healing, for instance vascular endothelial growth factor (VEGF), granulocytes-macrophage colony stimulating factor (GM-CSF) and transforming growth factor (TGF-β). However, these factors are regulated by IL-6 [23] and other cytokines measured in this study. Another limitation might be heterogeneity of patients’ population (CD and UC) and surgical procedures. However, sub-group analysis did not change the results which were in line with a recently published prospective study where the authors included only CD patients undergoing ileo-caecal resections [36]. The choice of anesthetic agents was left to the routine practice in the participating departments. This might be a limitation in the study as different anesthetic agents may affect the surgical stress response differently [37].

## Conclusion

This pilot study showed no difference in surgical stress response between anti-TNF-α treated and anti-TNF-α naïve patients. Withdrawal of anti-TNF-α drugs prior to surgical intervention in IBD patients might not be justified without measurement of drug concentration and drug antibodies. Further large sample prospective studies are needed.
